# Testicular seminoma presenting with duodenal perforation: a case report

**DOI:** 10.1186/1752-1947-2-294

**Published:** 2008-09-09

**Authors:** Ranko Miocinovic, Ronney Abaza

**Affiliations:** 1 Department of Urology, Dowling Hall 2nd floor, University of Toledo Medical Center, 3065 Arlington Avenue, Toledo, OH 43614, USA

## Abstract

**Introduction:**

Testicular neoplasms metastasizing to the retroperitoneum rarely involve the upper gastrointestinal tract. Gastrointestinal tract metastases usually present with complications including intestinal obstruction, gastrointestinal hemorrhage, and rarely ulceration of the bowel mucosa.

**Case presentation:**

We describe an unusual case of duodenal perforation as the presenting manifestation of metastatic classic type seminoma in a 45-year-old man.

**Conclusion:**

Germ cell tumor diagnosis should be considered when an ulcerating small bowel mass is identified in a young man.

## Introduction

Testicular germ cell tumor metastasis to the upper gastrointestinal (GI) tract is uncommon (<5%), and its likelihood is related to the histologic type of the primary neoplasm [1]. Non-seminomatous germ cell tumors are much more likely to spread to the gastrointestinal (GI) tract than seminomas [2]. In their series of 25 patients with GI tract involvement, Chait *et al. *reported the primary neoplasms to be combinations of embryonal carcinoma, teratoma, and choriocarcinoma [3]. The most commonly observed metastatic sites of the GI tract include the small intestine and duodenum [2,4]. Different modes of spread have been observed, but direct extension from the retroperitoneal lymph nodes is more frequent than peritoneal seeding or hematogenous spread [3]. GI complaints such as intestinal obstruction, volvulus, intussusception, and hemorrhage resulting from metastatic testicular neoplasms are the most common manifestations of the disease [2]. Although small bowel ulceration secondary to metastatic seminoma has been previously reported, it is very uncommon [1,5,6]. We report a case of classic type seminoma tumor presenting primarily as perforation of the duodenum and acute abdomen.

## Case presentation

A 45-year-old man presented with acute respiratory failure, hypotension, rigid abdomen, and a hard, erythematous, and tender 14 cm right inguinoscrotal mass. Initial differential diagnoses included incarcerated hernia and testicular cancer. Further information regarding the mass could not be elicited due to the patient's altered mental status and critical condition requiring immediate intubation. Plain abdominal radiograph was unremarkable and chest radiograph revealed two large pulmonary nodules suspicious for malignancy. The patient's laboratory findings revealed elevated lactate of 7.7 mmol/liter (normal 0.5–2.2), amylase of 518 units/liter (normal 20–128), lipase of 157 units/liter (normal 6–32), a normal WBC of 5.5 with a slight left shift, and normal tumor markers (alpha-fetoprotein and beta-HCG). He underwent an emergency exploratory laparotomy due to suspicion of an incarcerated hernia and was found to have succus entericus throughout the peritoneal cavity and significant para-aortic lymphadenopathy invading the second and third portion of the duodenum.

**Figure 1:**
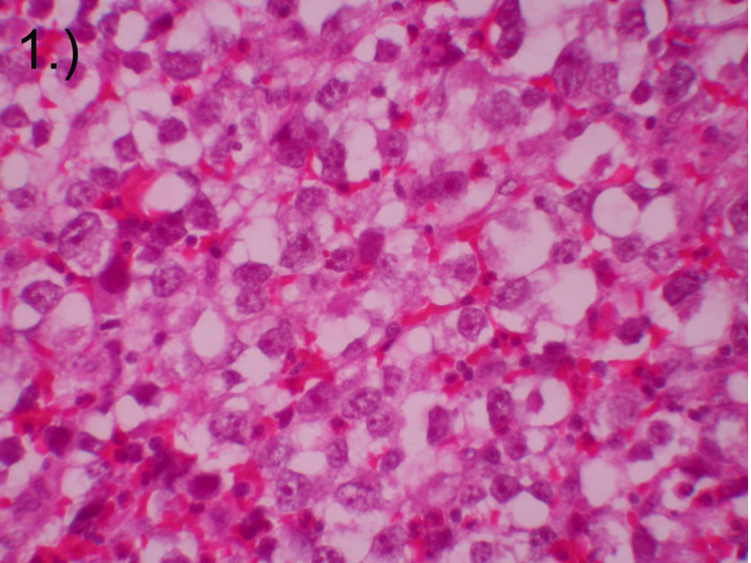
Hematoxylin and eosin stain of retroperitoneal mass biopsy consistent with classic seminoma. Magnification ×40.

**Figure 2:**
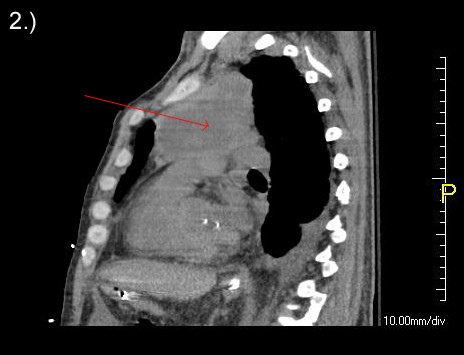
Computed tomography scan demonstrating extensive metastatic disease of the mediastinum.

**Figure 3:**
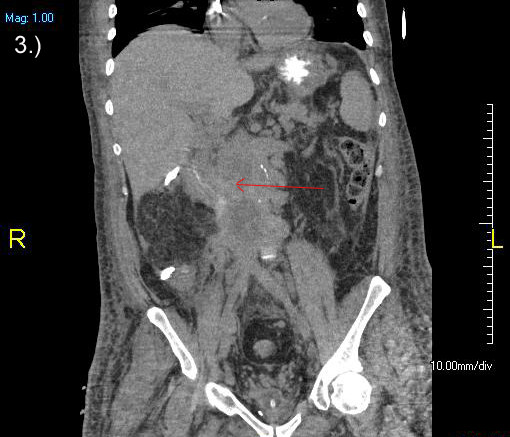
Computed tomography scan demonstrating extensive metastatic disease of the retroperitoneum.

**Figure 4:**
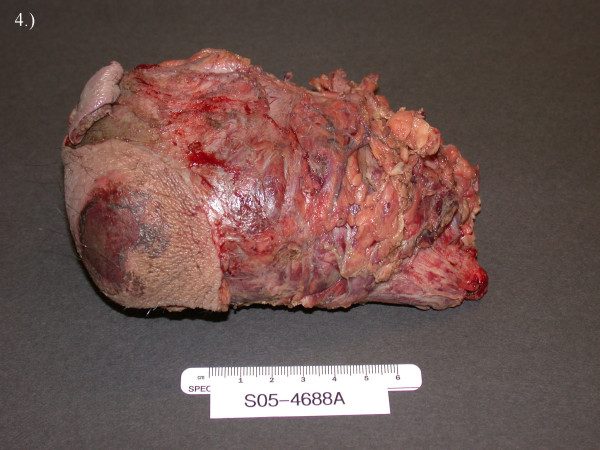
Gross specimen of testicular mass invading through the scrotum.

The perforated duodenal defect could not be repaired due to the extent of tumor infiltration. A controlled fistula was created with two separate red rubber catheters which were placed proximally and distally to the duodenal defect. A wedge tissue biopsy taken from the mass proved consistent with classic seminoma (Figure 1). The right scrotal mass was not explored during the initial operation due to the patient's critical condition in the operating room requiring postponement of surgery. On postoperative day three, a computed tomography (CT) scan was obtained and revealed extensive disease above and below the diaphragm (Figures 2 and 3). The scrotal mass eventually began to invade through the scrotal skin and required resection 1 week after initial surgery (Figure 4). The final pathology revealed seminoma, classic type, and the final staging was consistent with T3, N3, M1b disease.

The patient remained on respiratory support throughout his hospitalization and eventually succumbed to his disease 4 weeks later when his family declined further medical therapy. Treatment with chemotherapy was considered but not commenced due to the presence of the duodenal perforation and respiratory failure.

## Discussion

Involvement of the GI tract by metastatic seminoma is rare. When metastatic, only 5% of testicular germ cell tumors involve the bowel, and seminoma is the least commonly reported type of testicular neoplasm to do so [1]. In an autopsy study by Johnson *et al.*, pure seminoma was the histologic type observed in only two of the 21 metastatic germ cell tumors identified in the GI tract [4]. Whereas the small bowel was the most common GI site of involvement in their study, the duodenum was only rarely involved. A series of 487 postmortem cases evaluated by Chait *et al. *did not document any purely seminomatous tumors metastasizing to the GI tract [3]. In a review article by Sweetenham *et al.*, three cases of seminoma metastatic to the duodenum and stomach were described, with severe upper abdominal and back pain having been the predominant symptoms in all patients [2]. The involvement of the duodenum by metastatic testicular neoplasms has been attributed to the position of its second, third and fourth segments in the retroperitoneum where the lymphatic drainage of the testis is located [2].

A case of an ulcerating retroperitoneal seminoma mass communicating with the distal duodenum has been reported previously [5]. However, the patient was cured successfully with several courses of chemotherapy and non-surgical management. The authors note that, although other cancers such as melanoma, kidney, and stomach are much more common than germ cell tumor (GCT) and more often metastasize to the small bowel, this diagnosis should be considered when an ulcerating small bowel mass is identified in a young man [5].

## Conclusion

Although modern chemotherapeutic regimens have high success rates in the treatment of metastatic seminoma, some authors have proposed that patients with intestinal metastasis belong to a high-risk group [3,7]. Early intervention with surgical resection of the involved segment of bowel is necessary when GI complications are encountered. This group of patients with intermediate or high-risk traditionally receive four cycles of bleomycin, etoposide, and cisplatin (BEP) with a known 30% to 40% failure of achieving a durable response [7]. Unfortunately, in our patient with multiple complicating medical factors secondary to a late stage seminoma with bowel perforation, the chemotherapy was never initiated and the patient died.

## Competing interests

The authors declare that they have no competing interests.

## Consent

Written informed consent could not be obtained since the patient is deceased and the next of kin were untraceable. We believe this case report contains a worthwhile clinical lesson which could not be as effectively made in any other way. We expect that the next of kin would not object to the publication since the patient remains anonymous.

## Authors' contributions

Both authors contributed equally in preparing this document and were equally involved in care of the patient. RM was responsible for writing the introduction and case report itself, while RA contributed to the conclusion section and revision of the final document. Both authors were involved in creating CT and pathology images.
